# Identification of universal and cell-type specific p53 DNA binding

**DOI:** 10.1186/s12860-020-00251-8

**Published:** 2020-02-18

**Authors:** Antonina Hafner, Lyubov Kublo, Michael Tsabar, Galit Lahav, Jacob Stewart-Ornstein

**Affiliations:** 1 Department of Systems Biology, Harvard Medical School, Boston, MA 02115 USA; 2 Department of Developmental Biology, Stanford University, Stanford, CA 94305 USA; 3 University of Pittsburgh Medical Center (UPMC) Hillman Cancer Center, University of Pittsburgh School of Medicine, Pittsburgh, PA 15213 USA; 4 Department of Computational and Systems Biology, University of Pittsburgh Medical School, Pittsburgh, PA 15260 USA

**Keywords:** p53, ChIP-seq, Chromatin, DNA damage, Gene expression

## Abstract

**Background:**

The tumor suppressor p53 is a major regulator of the DNA damage response and has been suggested to selectively bind and activate cell-type specific gene expression programs. However recent studies and meta-analyses of genomic data propose largely uniform, and condition independent p53 binding and thus question the selective and cell-type dependent function of p53.

**Results:**

To systematically assess the cell-type specificity of p53, we measured its association with DNA in 12 p53 wild-type cancer cell lines, from a range of epithelial linages, in response to ionizing radiation. We found that the majority of bound sites were occupied across all cell lines, however we also identified a subset of binding sites that were specific to one or a few cell lines. Unlike the shared p53-bound genome, which was not dependent on chromatin accessibility, the association of p53 with these atypical binding sites was well explained by chromatin accessibility and could be modulated by forcing cell state changes such as the epithelial-to-mesenchymal transition.

**Conclusions:**

Our study reconciles previous conflicting views in the p53 field, by demonstrating that although the majority of p53 DNA binding is conserved across cell types, there is a small set of cell line specific binding sites that depend on cell state.

## Background

p53 is the major transcription factor regulating the DNA damage response in mammals, by inducing transcription of genes involved in DNA repair, cell cycle arrest and apoptosis{Kruiswijk, 2015 #3} [1, 2]. Though ubiquitously expressed across human tissues, it remains unclear to what extent p53 functions are shared across different cell types. Context specific regulation of gene expression by p53 has been a long-standing hypothesis in the p53 field, and implies that p53 can integrate information about cellular context and the type of stress to selectively activate some target genes versus others [1, 3–5]. Several studies have shown that there are cell-type specific p53 DNA binding sites and corresponding activation of gene expression [6–10]. However, comparison of p53 binding across multiple cell in different human cell lines or upon different treatments has shown a strong agreement in the majority p53 binding locations [10, 11] and activation of a core set of target genes [12]. These studies compared pairs of cell lines or supplemented single cell line data with meta-analysis of published datasets, an approach that is powerful for identifying universal p53 binding sites but has limits for detection of cell line specific binding patterns due to divergent experimental conditions across datasets.

In this work, we explored cell-type and stimulus specificity of the tumor suppressing transcription factor p53 at the level of DNA binding. To study how p53 binding varies across cell types, we measured p53 DNA binding in 12 cancer cell lines from different tissue types in which we have previously shown a comparable accumulation of p53 [13] in response to ionizing radiation (IR). By treating this panel of epithelial cell lines with a dose of IR sufficient to induce uniform p53 activation across cell lines and measuring p53 binding at an early (2 h) time-point we minimized secondary effects and focused on measuring the rapid and direct binding of p53. Our approach differs from the majority of p53 datasets in the literature, which use chemotherapy agents such as doxorubicin or the p53 activator Nutlin3A at later time-points of 6 to 12 h. This coherent set of samples allowed us to rigorously explore the heterogeneity of p53 binding and identify the influence of universal genomic and cell line specific chromatin factors on p53 binding.

We found that the majority of p53 binding events to be universal across cancerous cell lines and RPE1, a non-cancerous transformed line, with strong quantitative agreement in binding magnitude. We further found that Nutlin3A treatment resulted in a nearly identical set of p53 binding events as IR, suggesting the conservation of these binding sites across treatments [11]. The presence of highly conserved p53 DNA binding sites is consistent with previous meta-analysis of p53 DNA binding [10, 11]. However, we also identified a set of variable p53 binding events (~ 5%) present in only one or a handful of cell lines. These binding events were often near transcriptionally active genes and correlated strongly with cell line specific chromatin accessibility. Consistent with this, we were able to alter p53 DNA binding when we pharmacologically modified the chromatin state or induced an epithelial-to-mesenchymal transition to globally change cell state. Taken together, our data shows that the majority of p53 DNA binding is context independent but there is a small but potentially important set of cell-type or cell-state specific binding sites for p53.

## Results

### p53 binding across the genome is stereotyped across cell lines

**Fig. 1:**
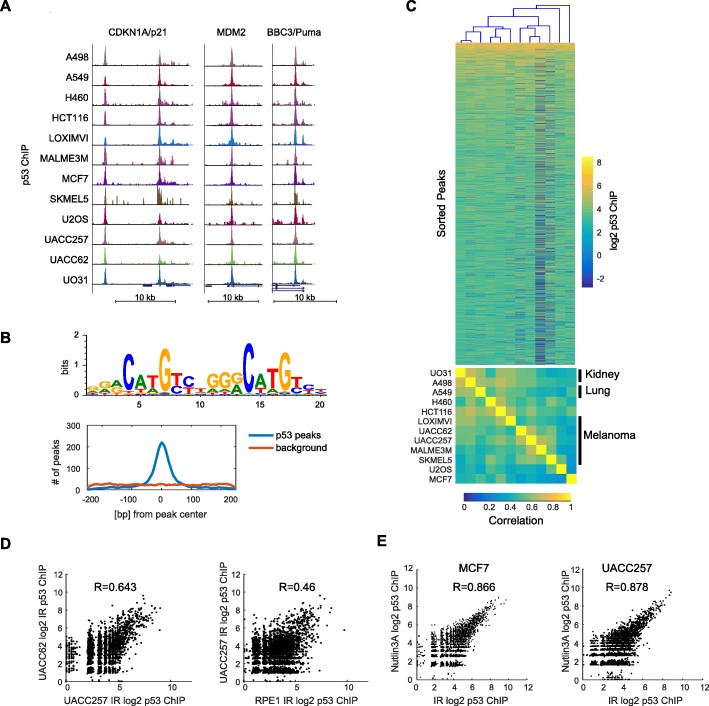
Stereotyped p53 binding across 12 cell lines. (**A**) ChIP-seq for p53 in 12 p53 wild-type cell lines. UCSC screen shots of p53 binding sites for three canonical p53 target genes are shown. (**B**) Motif analysis recovered a p53 motif that was centrally enriched within peaks. (**C**) Heatmap showing p53 binding intensity in 8742 locations in the genome. Cell lines were clustered on p53 binding and resulted in lineages clustering together. (**D**) Comparison of p53 binding in two cancer cell lines (UACC62 and UACC257) as well as between one cancer (UACC257) and one non-cancerous cell line (RPE1). (**E**) Comparison of p53 binding between Nutlin3A and IR treated samples in MCF7 or UACC257 cells

To study how p53 binding varies across cell lines we treated 12 cell lines expressing wild type p53 with ionizing radiation (IR; X-Ray 4Gy) for 2 h and performed ChIP-Seq. We have previously shown that these cell lines show similar (with 2-fold) p53 abundance at this time point [13]. Visual inspection of well-established p53 target genes showed clear ChIP peaks in all cell lines (Fig. 1a). Overall, by pooling data from all cell lines, we confidently called 8742 p53 ChIP peaks. De novo motif analysis identified the p53 binding motif that was centrally enriched within peaks (Fig. 1b) and closely matches the experimentally validated binding site [14].

The quantitative strength of p53 binding at each genomic locus was highly conserved across the 12 cell lines (Fig. 1c). Aside from weaker p53 signal in the SKMEL5 and MALM3E cell lines, no strong groups of cell lines appeared by eye. However, hierarchical clustering sorted the cell lines by tissue of origin, with pairs of lung and kidney lines, and melanoma lines grouping together (Fig. 1c). These p53 bound regions were also similar to other published datasets [11] (average within dataset Pearson correlation 0.53+/− 0.099 (stdev), average correlation to external datasets 0.41+/− 0.11; Additional file 4: Figure S1). It was previously suggested that cancer cell lines show a different p53 binding profile from non-cancerous cells [6]. We therefore compared the 12 cancer cell lines to an identically treated non-transformed line, RPE1, which we treated with IR, identically to the cancer cell lines. We found that p53 binding at identified sites in RPE1 cells in response to IR was highly correlated with p53 binding in the 12 cancer cell lines (Fig. 1d; average Pearson r = 0.48+/− 0.117 for correlation (RPE, Cancer Lines) vs an average of 0.53+/− 0.099 for correlation (Cancer, Cancer)).

To further explore if the apparent uniformity of p53 binding is specific to IR, we treated, two cell lines, MCF7 and UACC257, with a small molecule, Nutlin3A, that is known to activate p53{Vassilev, 2004 #37} [15]. In both MCF7 and UACC257, 2-h treatment with IR or Nutlin3A lead to similar levels of p53, with Nutlin3A producing slightly higher amounts (Additional file 4: Figure S2). Comparison of p53 ChIP peaks between different conditions and cell lines, showed that IR-Nutlin3A correlations within each line that were stronger than any line - line correlations (Fig. 1e, Pearson r = 0.87 or 0.88 for MCF7 and UACC257, respectively, vs r = 0.73 for the maximum line-line) and is consistent with recent work showing clustering of p53 DNA binding by cell type and not treatment [16]. Thus, IR induced and pharmacologically induced p53 do not lead to distinct p53 function as measured by acute p53 DNA binding, as is consistent with recent work [17]. Overall, our data shows that p53 DNA binding is globally conserved across cell types and treatments, however clustering of cell types by tissue or origin suggests that there may be p53 DNA binding features that are cell-type specific.

### Genomic DNA sequence has limited predictive power for p53 binding strength

**Fig. 2:**
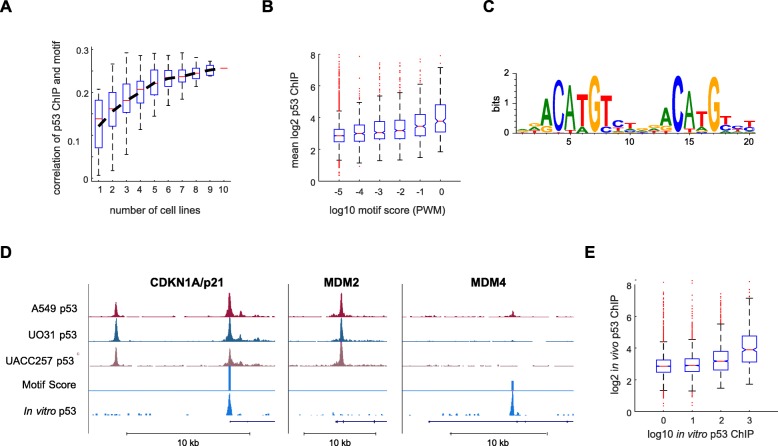
Genomic sequence is weakly predictive of p53 binding. (**A**) The correlation between motif strength and p53 binding is shown as a function of the number of cell lines across which the peak height was averaged, box plots represent the distribution of correlations across all possible cell line combinations. (**B**) The degree to which p53 binding motif predicts the strength of p53 binding is shown in a box plot, with p53 binding sites binned by their motif strength. (**C**) The top enriched motif identified by in vitro ChIP. (**D**) UCSC screenshots of p53 binding sites in A549, UO31 and UACC257 in response to IR, motif score, and in vitro p53 binding signal are shown for CDKN1A/p21, MDM2, and MDM4. (**E**) In vivo p53 binding strength is shown in a box plot, binned by in vitro p53 binding signal at each genomic site

Given the strong conservation of p53 binding across cell lines, and the recent analyses showing that DNA sequence is the best predictor of genomic p53 binding [11] we wondered if the DNA sequence was predictive of p53 binding strength. We tested this by comparing motif scores (calculated from the position weight matrix (PWM)) with p53 ChIP-seq signal intensity. The extent of the correlation between p53 ChIP signal and PWM score was highly cell line dependent (Fig. 2a), ranging from no correlation to correlation of 0.22 in a single cell line. Averaging p53 binding over increasing numbers of cell lines resulted in better agreement between genomic motif score and p53 binding, with the highest correlation being 0.26, when we averaged across all datasets (Fig. 2a, b). Therefore, although the motif score significantly correlates with p53 DNA binding (Pearson’s r = 0.26, *p* = 2.0e-132), it only accounts for ~ 6% of the variance.

To explore if our motif analysis was simply a poor model of p53 binding, we performed an in vitro ChIP experiment. In this experiment, recombinant p53 was incubated with fragmented genomic DNA. This was followed by immunoprecipitation and deep sequencing, similarly to a recently published protocol [18]. As this assay uses fragmented protein free genomic DNA (with a size of ~ 300-600 bp), effects of chromatin or other factors that may influence in vivo p53 interaction with DNA, should not be present. We obtained a strong signal of p53 binding which was reproducible between replicates (Additional file 4: Figure S3A, B), recovering a consensus p53 motif (HOMER *p* = 1e-2422, Fig. 2c), very similar to the motif found in vivo (Fig. 1b)*.* We observed p53 binding sites, such as the one proximal to the CDKN1A/p21 promoter, that showed strong in vivo binding, a strong motif, and substantial in vitro p53 binding (Fig. 2d). Surprisingly, other binding sites, such as the one contained in the first intron of MDM2, showed substantial in vivo binding, but little in vitro binding and no strong motif. Conversely, the binding site at the MDM4 gene showed strong in vitro binding and a strong motif, but little in vivo binding. Overall, the in vitro p53 binding signal did not show a better correlation (Pearson’s r = 0.25, *p* = 3.1e-127, Fig. 2e) with in vivo p53 binding than the motif score. Although we note this correlation combines two datasets susceptible to measurement noise (in vitro and in vivo ChIP-seq) may underestimate this correlation. These results suggest that factors other than DNA sequence determine p53 binding in vivo.

### A subset of p53 binding sites are cell-type specific

**Fig. 3:**
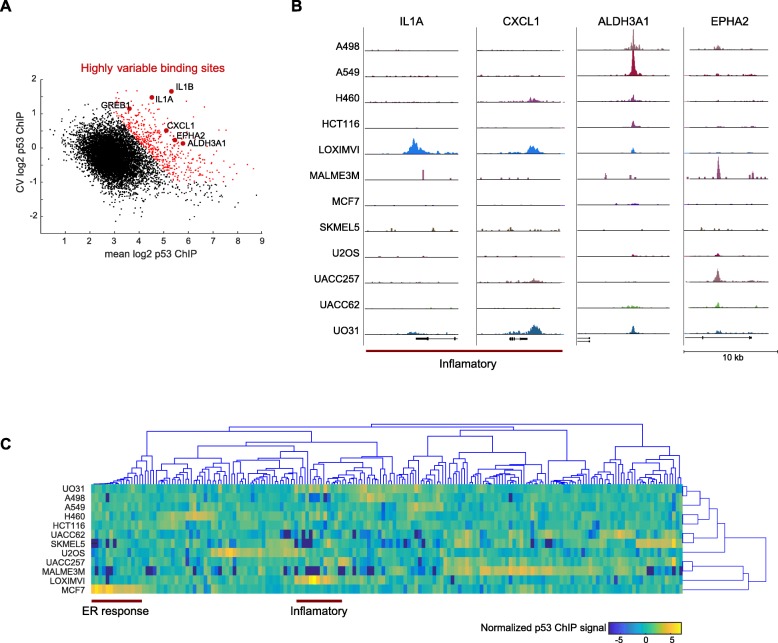
Variable p53 binding sites show cell-type specific functional enrichment. (**A**) Scatterplot of all 8742 p53 binding sites in response to 4Gy IR by their average ChIP signal and coefficient of variation (CV). Highlighted in red are ‘highly variable’ peaks defined as having higher than expected CV relative to the peak height. Example binding sites are labeled with the associated gene names. (**B**) UCSC screen shots of four example ‘variable’ peaks. (**C**) Heatmap of ‘variable’ p53 peaks that are also nearby (< 10 kb) transcription start sites of genes. The intensity of each peak is normalized to the average across 12 cell lines. Cell lines and peaks were hierarchically clustered, with no grouping by lineage observed for cell lines. Groups of inflammatory and the ER associated are highlighted

Our finding of a uniform set of p53 bound regions independent of cell line or even treatment is consistent with previous work [11]. However, clustering of cell types by tissue of origin (Fig. 1c), made us wonder if we could also find cell-type specific p53 binding that, due to the uniformity of our dataset (both in treatment and data collection) and early time-point of treatment, might have been missed in earlier analyses. We compared the cell line to cell line variability in p53 ChIP signal after correcting for the average ChIP peak signal (which contributes shot noise to our analysis) and identified about 5% of peaks (494 peaks) that showed high variation between cell lines relative to their average peak strength (Fig. 3a, b). For example, p53 peaks nearby the inflammatory associated genes IL1A and CXCL1 showed clear p53 binding in the LOXIMVI line, weaker association in the UO31 and H460 lines, and no binding in other cell lines (Fig. 3b). We also found variability in p53 binding at the promoters of previously reported p53 target genes, ALDH3A1 and EPHA2, ranging from no binding in some cell lines to strong peaks in others (Fig. 3b). De novo motif search on this set of variable peaks identified the p53 binding site as the most significantly enriched motif (HOMER, *p* = 1.0e-46), suggesting that these sites represent direct p53 binding events.

To determine if these highly variable binding sites had novel cell line specific functions, we selected peaks that mapped within 10 kb of transcription start of genes, resulting in 218 peaks. We found that most cell lines showed a few unique p53 binding peaks, but without strong clustering between cell lines (Fig. 3c) as in Fig. 1c. Enrichment analysis identified inflammatory/chemotaxis associated genes as being enriched in these highly variable p53 bound genes. The cell line LOXIMVI showed particularly strong enrichment for p53 binding to inflammatory genes including IL1A, IL1B, CLL20, and CXCL1. UO31 also showed substantial binding for many of these targets. We also observed, that in the estrogen receptor (ER) positive MCF7 breast cancer cell line, several MCF7-specificp53 peaks overlapped with ESR1 (estrogen receptor) binding sites, and were in proximity of genes such as TFF1, IGFBP4, and PRLH. These results suggest that the cell-type specific p53 binding sites we discovered may be linked to cell line specific regulatory programs.

### Cell line specific chromatin accessibility accounts for variability in p53 binding sites

**Fig. 4:**
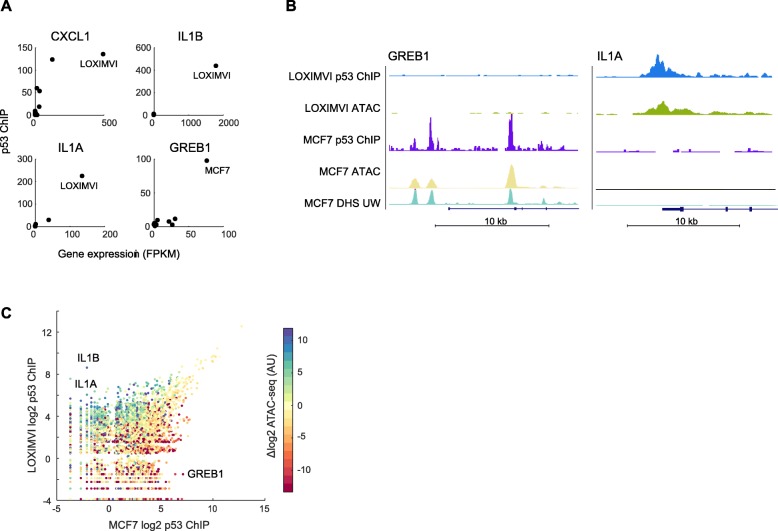
Chromatin accessibility contributes to variable p53 binding. (**A**) Scatterplots illustrating the relationship between basal gene expression and p53 ChIP signal the 12 cell lines for four p53 peaks/genes. Note that in many cases, multiple cell lines show little gene expression or p53 binding and therefore cluster near the origin. (**B**) UCSC screen shots of two p53 binding sites; p53 binding in the proximity of GREB1 is found in MCF7 treated with IR, while IL1A was bound in IR treated LOXIMVI cells. ATAC-seq data and published DNase hypersensitivity data (for MCF7, untreated) showing that IR induced p53 binding correlates with basal DNA accessibility in each cell line. (**C**) Scatter plot of p53 binding post IR in MCF7 compared to LOXIMVI, colored by the difference in ATAC-seq signal: log2(LOXIMVI)-log2(MCF7) between the two cell lines

The differences we observed between in vivo and in vitro DNA binding and the presence of cell-type specific p53 binding cannot be explained by the motif. We thus hypothesized that chromatin accessibility may play a role in tuning in vivo p53 DNA binding. Consistent with this hypothesis, we observed a significant relationship of cell-line specific p53 peaks with basal gene expression (two-sided t-test, *p* = 1.9e-31, Additional file 4: Figure S4), that we measured by RNA-seq. For example, basal mRNA expression of IL1A, IL1B, CXCL1, and GREB1 were all associated with p53 binding across the 12 cell lines (Fig. 4a). In contrast, the fold change induction of gene expression in response to IR (3-h time-point) was uncorrelated with p53 ChIP signal both for cell-line specific p53 ChIP peaks and for established p53 target genes [12] (Additional file 4: Figure S4, list of target genes in Additional file 3: Table S3) consistent with other studies [7, 16, 19]. Indeed, even the most canonical target genes, CDKN1A, MDM2 and BBC3, showed variable induction across cell lines despite conserved p53 binding at these genes in all 12 cell lines (Fig. 1a, Fig. S4). Our results linking basal expression of nearby genes to p53 binding suggest that the ‘openness’ of the genomic region might influence p53 binding, which is consistent with the previous observations that p53 binds readily in open regions [20–22].

To directly measure the connection between chromatin accessibility and DNA binding, we performed ATAC-seq. We chose two cell lines, LOXIMVI, which showed strong, and unique binding of p53 nearby inflammatory related genes and MCF7, which showed p53 binding at estrogen receptor associated genes. We performed a modified ATAC-seq protocol using the MuA transposase to generate genome wide maps of accessible regions in the MCF7 and LOXIMVI cell lines. Our ATAC-seq data and ENCODE produced DNAse sensitivity data from MCF7 showed strong overlap with greater than 90% of ATAC-seq peaks being DNAse accessible [23]. We compared our ATAC-seq data to the p53 ChIP-seq signal for the inflammatory genes that showed p53 binding in LOXIMVI but not in MCF7 and observed strong ATAC-seq signal only in the LOXIMVI cell line (Fig. 4b), consistent with increased accessibility at these loci leading to stronger p53 binding. Conversely, GREB1, a breast cancer associated gene showed only p53 binding and ATAC-seq signal in MCF7 cells (Fig. 4b). Moreover, genome wide, the difference in ATAC-seq signal between the two lines accounted for 22% of the variance in p53 binding between the two datasets (R^2^ = 0.225; Fig. 4c). More generally, as observed for other transcription factors [24], combining accessibility and motif scoring allows for improved prediction of DNA binding. Indeed, accessibility and motif score accounted for 13.8 and 20.9% of the variance in the log2(p53 ChIP-seq peak signal) for MCF7 and LOXIMVI respectively, compared to ~ 6% with the motif alone. We therefore conclude that chromatin accessibility favors p53 binding and accounts for a substantial fraction of the cell line specific gain of p53 DNA binding sites between MCF7 and LOXIMVI cells. Interestingly, we also found that genome wide chromatin accessibility was negatively correlated with in vitro p53 binding (Pearson’s r = − 0.2, *p* = 2.1e-80, MCF7 ATAC-seq vs. in vitro binding), suggesting that many strong p53 binding sites are obscured by local chromatin context.

### Perturbation of cell state alters p53 DNA binding

**Fig. 5:**
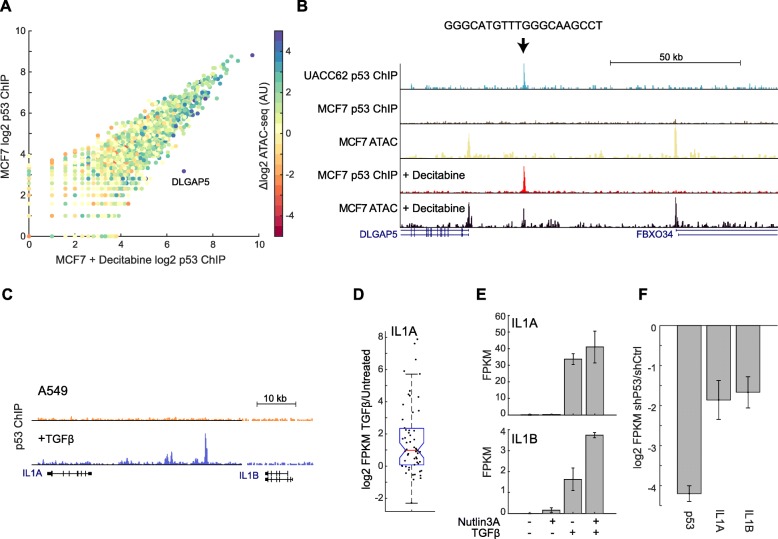
Cellular state regulates p53 binding. (**A**) Scatter plot of p53 binding in IR treated MCF7 cells compared to MCF7 treated with IR and decitabine, colored by the difference in ATAC-seq signal between decitabine treated and untreated cells: log2(decitabine)-log2(untreated). (**B**) UCSC screen shot of the region around the gene DLGAP5, showing changes in p53 binding and accessibility in the decitabine treated MCF7 cells (the new peak is indicated by an arrow). Binding of p53 in IR treated UACC62 cells at the DLGAP5 locus without decitabine treatment is also shown. (**C**) UCSC screen shot of the IL1 locus showing increased p53 binding in TGFβ treated cells. (**D**) Boxplot showing log2 change in gene expression (TPM of TGFβ treated/untreated) in genes nearby p53 binding sites that showed altered occupancy in TGFβ treated cells. (**E**) Gene expression of IL1A and IL1B in cells treated as indicated with Nutlin3A or TGFβ (*N* = 3 experiments, except TGFβ + Nutlin3A *N* = 2). (**F**) Expression of IL1A and IL1B measured by RNA-seq in A549 cells treated with TGFβ comparing p53 knockdown cells to control knockdown (*N* = 3). All error bars are SEM

To establish a causal link between chromatin state and p53 binding, we treated MCF7 cells with decitabine, a methylase inhibitor that has been shown to broadly alter chromatin structure [25]. We then treated these cells with IR and preformed p53 ChIP-seq and ATAC-seq. Comparing p53 binding between the decitabine treated and untreated cells, showed a modest but significant correlation between change in chromatin accessibility and change in p53 DNA binding between decitabine treated and untreated samples (Pearson’s r = 0.16, *p* = 3.99e-13). Looking at differential peaks between conditions, we found only one binding site, adjacent to the DLGAP5 gene, that showed a substantial change in p53 binding (Fig. 5a). This increase in p53 binding was accompanied by increased accessibility (Fig. 5b). The DLGAP5 binding site has a consensus p53 motif and showed occupancy in other cell lines such as UACC62 (Fig. 5b). Overall, these data show that decitabine treatment results in chromatin changes that can favor p53 binding at some binding sites but does not alter the global p53 DNA binding profile (Fig. 5a), perhaps due to limited overlap of accessibility changes and p53 binding sites.

We next asked whether a more dramatic perturbation of chromatin accessibility and cellular state can alter the cell-type specific p53 DNA binding sites. The LOXIMVI cell line shows p53 binding at inflammatory genes such as IL1, that we were unable to induce with decitabine treatment in MCF7 cells. We noted that the LOXIMVI line has been previous reported to have a mesenchymal phenotype [26]. We wondered if forcing an Epithelial to Mesenchymal Transition (EMT) on another cell line would result in changes to p53 binding? A549 cells have been shown to undergo EMT when treated with TGFβ [27], we therefore treated A549 cells with TGFβ for five days to induce EMT and measured p53 binding with ChIP-seq. We observed many new binding sites for p53, particularly we noted the emergence of p53 binding at the IL1A/B locus (Fig. 5c). We also see a similar peak at the IL1A locus in the published p53 ChIP-seq data in the CAL51 cell line which is classified as mesenchymal (Additional file 4: Figure S5). In the A549 cells treated with TGFβ, novel or stronger binding in the genome (2 std. dev. above untreated) was associated with increased expression of nearby genes under basal p53 condition (Fig. 5d). For IL1A and IL1B, we noted increased expression under both basal p53 and Nutlin3A induced p53 (Fig. 5e). Expression of IL1A and IL1B was partially dependent on p53 as knockdown of p53 reduced expression of these genes (Fig. 5f), this was also true for the LOXIMVI line (Additional file 4: Figure S6) which naturally expresses IL1A/B and has associated p53 binding at these genes. Therefore, cell state affects p53 DNA binding and regulation of target genes including inflammatory genes.

## Discussion

The transcription factor p53 regulates the cellular response to DNA damage, including up-regulating repair, cell cycle arrest and apoptotic proteins. The nature, strength, and balance between the DNA repair and cell death arms of p53 signaling varies across tissues in the body [3, 13, 28], and can be modified by drug treatment [29, 30] and genetic perturbation [31]. The role of the p53 itself in this decision making is controversial, with arguments for p53 behaving as a smart ‘signal integrator’ (reviewed in [1]) or a simple effector [11]. We sought to understand the role of p53 in diverse cell lines by focusing on p53 DNA binding and gene expression in response to ionizing radiation.

To date, there has been a number of studies that measured p53 DNA binding genome-wide. Depending on the specific focus of the study, the conclusions vary greatly in either emphasizing cell-type specific events or concluding that p53 DNA binding is independent of cell context or treatment. A recent study, supporting the latter conclusion, also argues that the cell-type specific binding events were indirect or non-functional [11]. In order to understand to what degree p53 DNA binding is either conserved or specific to cellular context, we chose to collect p53 ChIP-seq data across 12 cell types from various tissues of origin and an early time-point post DNA damage to avoid long term and indirect effects of DNA damage. We found a large degree of conservation in p53 binding, however saw that p53 binding could group cell lines by their tissue of origin, suggesting some degree of tissue specificity consistent with [16]. Taking advantage of the coherence of our dataset we identified p53 binding sites that were variably occupied across cell lines. This subset of peaks were nearby genes enriched for specific cellular programs, most notably the inflammatory response in the melanoma LOXIMVI cell line and ER specific response in the MCF7 cell line.

Further, we noted a modest, but significant correlation between the strength of p53 binding (measured by ChIP-seq) and the predicted strength of p53 association (p53 motif). This correlation varied across cell lines and was strongest in the pooled dataset containing all cell lines. More strikingly, we observed a similar correlation when comparing genome wide in vitro association of p53 with in vivo p53 binding. In general, p53 binding at any given location in the genome was relatively poorly predicted by either in vitro binding or motif analysis suggesting that in vivo factors greatly contribute to p53 binding specificity.

We found that chromatin accessibility explains a significant amount of the differential p53 binding between the MCF7 and LOXIMVI cell lines. Globally, our data showed that a higher degree of chromatin accessibility favored p53 binding adding to the complex litterature on chromatin-transcription factor associations [32, 33]. We observed strong p53 binding to inflammatory genes in the LOXIMVI cell line and also in the TGFβ induced A549 line. Expression of these inflammatory genes was partially dependent on p53 (Fig. 5, Additional file 4: Figure S6). These results mirror an emerging role for p53 in inflammatory gene regulation in macrophages [34] and fibroblasts [35]. Depending on the degree and context in which p53 drives these inflammatory signaling, this may position p53 as a regulator of inflammatory signaling in epithelial systems including many cancers.

## Conclusions

Taken together, our results suggest there may be two classes of p53 binding sites that are not clearly distinguished by p53 binding motif, that the majority of sites, that are invariably bound across cell types and treatments, and ~ 5% of the sites, that are cell type specific and in contrast to the latter, require accessible chromatin or other auxiliary factors to function. Supporting a mixed model of partial dependence of p53 on the cellular state to regulate its binding, we showed that alteration to the cellular state either using pharmacological agents targeting chromatin or the endogenous ligand TGFβ to alter cellular state resulted in substantial changes to p53 binding. Further studies coupling chromatin accessibility, chromatin state, p53 binding, post-translational modifications, and measurements of RNA synthesis and degradation rates will be required to reconcile different models of p53 regulation and identify what features tune the cellular response to DNA damage in different cellular backgrounds.

## Methods

### Cell culture and cell treatment

The following cancer cell lines are part of the NCI-60 collection (https://dtp.cancer.gov/discovery_development/nci-60/cell_list.htm): A549, A498, H460, HCT116, LOXIMVI, MALME3M, MCF7, SKMEL5, UACC257, UACC62, UO31. All parental cell lines, with the exception of RPE1 cells (gift from Prof. Steve Elledge, Harvard Medical School), were obtained from ATTC: A498 (ATCC HTB-44), A549 (ATCC CCL-185), H460 (HTB-177), HCT116 (CCL-247), SKMEL5 (ATCC HTB-70), U2OS (HTB-96), MCF7 (ATCC HTB-22), LOXIMVI, MALME3M, UACC257, UACC62, UO31. Cells were thawed and propagated in RPMI (GIBCO) with 5% FBS. All experiments were performed in this media. All media was supplemented with 1% antibiotic and antimycotic (Corning). Treatment with Nutlin3A (Sigma) was at 5 μM. X-ray induced DNA damage was generated with a RS-2000 source (RadSource, 160KeV). MCF7 cells were treated with 2 μM decitabine (5-AZA-2′-deoxycytidine, MP Biomedicals) for 5 days, cells were split on day 2, re-plated in decitabine containing media. Treated and untreated cells were then further treated with IR or not as with other samples. A549 cells were induced to undergo epithelial-to-mesenchymal transition by treatment with TGFβ (Sigma) at 2.5 ng/ml for 5 days. For knockdown of p53, A549 cells were infected with a doxycycline inducible p53sh [36], selected on puromycin for infected cells. Subsequent induction of doxycycline was for 24 h with 500 ng/ml (sigma).

### ChIP-seq

p53 ChIP-seq was performed largely as previously described [37], briefly, 10 M cells were treated with 4Gy IR (RS-2000, RadSource) and 2 h later were fixed by addition of 1% paraformeldehyde (Alfa Aesar) at room temperature for 10 min with agitation. Fixation was stopped by addition of 250 mM glycine. Cells were scraped and flash frozen. Cell pellets were thawed in hypotonic lysis buffer and spun to generate a crude nuclei prep. These nuclei were lysed in an SDS buffer and sonicated (Bioruptor) to fragment DNA. Fragmented DNA was diluted in IP buffer and agitated overnight with 2 mg/ml DO-1 (anti-p53, Santa Cruz). 20 μl of protein A magnetic beads (Invitrogen) were used to isolate the p53 associated fragments and samples were washed with low salt, high salt, and LiCl buffers. DNA was eluted from beads with an SDS/NaCO3 buffer and was de-cross-linked at 65C for 6 h in a high salt buffer.

For experiments in Fig. 5, ChIP-seq was preformed using a Micrococcal Nuclease protocol. Briefly, cells were fixed and nuclei extracted as above, DNA was fragmented by a 20-min incubation with Micrococcal Nuclease (NEB) at 37C. Nuclei were then lysed by brief sonication (Branson) and fragmented DNA were Immuno-precipitated as described above.

ChIP libraries were constructed with the commercial NEBnext kit (NEB) and associated protocols, although reaction volumes were reduced by 4-fold and custom adaptors and barcodes were employed. Libraries were sequenced with single end 75 bp reads on Illumina NextSeq 500.

### In vitro ChIP-seq

To generate recombinant p53 we in vitro transcribed/translated human p53 with a c-terminal HA tag using a rabbit reticulocyte system (Promega). To generate fragmented genomic DNA we tagmented 50 ng of human genomic DNA from MCF7 cells using the MuSeq kit (Thermo) and amplified it using PCR and custom adaptor primers for 8 cycles. DNA was cleaned up on SPRI beads (Aline Biosciences) and quantified. At room temperature 20 ng of DNA and recombinant p53 (0.1 μM final) were combined in a binding buffer (10 mM TRIS, 5 mM MgCl2, 10% glycerol, 1 mM DTT) and incubated at room temperature for 30 min. The mixture was diluted 2-fold (to 20 μl) and 1.5 μl of anti-HA antibody was added (Rockland) and the sample incubated at 4C overnight with shaking. A 1:1 mixture of magnetic proteinA/proteinG beads was added (Sigma) and incubated at 4C for 1 h with shaking. The beads were then washed 3x with washing buffer (10 mM Tris, 5 mM HCL, 0.1% triton, 150 mM NaCl) and DNA eluted with elution buffer (1%SDS, 100 mM Na2CO3) at 37C for 15 min. Samples were cleaned up, and adaptors and barcodes added by PCR. Reads (> 30 M) were trimmed to remove adaptors with cutadapt [38], aligned to the genome with Bowtie, and analyzed with Matlab.

### RNA-seq

For each cell line 50,000 cells were plated in 35 mm dishes, 24 h later cells were treated (or not) with 4Gy IR (RS-2000, RadSource), 3 h after that cells were lysed with Trizol (Ambion). RNA was purified on affinity columns and DNAse treated (Zymo). Purified RNA (500 ng) was polyA purified using magnetic beads (NEB), fragmented and reverse transcribed using protoscript RT (NEB), second strand synthesized (NEB), and then assembled into libraries with the commercial NEBnext kit (NEB) and associated protocols, although reaction volumes were reduced by 4-fold and custom adaptors and barcodes were employed. Libraries were sequenced with single end 75 bp reads on a NextSeq.

### ATAC-seq

ATAC-seq was performed as described [39], with the major exception of the use of a MuA transposase (Thermo) rather than the TN5 transposase. Briefly, MCF7 or LOXIMVI cells were trypsinized and 50 K cells, spun down, washed once with PBS, and lysed with a hypotonic buffer containing 0.1% NP-40, and spun down to generate a crude nuclei pellet. This pellet was transposed in a 30 μl volume using MuA (0.7 μl), MuA buffer (10 μl), and H2O (19 μl) for 5 min at 30C. The sample was treated with 3 μl stop solution, and incubated at 30C for a further minute. The sample was then collected and purified by addition of 45 μl of SPRI beads (Aline Biosciences). The purified sample was PCR amplified in two steps to add barcoded adaptors suitable for Illumina sequencing. Samples were sequenced with single end 75 bp reads on an Illumina NextSeq. Reads (> 30 M) were trimmed to remove adaptors with cutadapt [38], aligned to the genome with Bowtie, and analyzed with Matlab. Genomic DNA (50 ng) from MCF7 and LOXIMVI was transposed, amplified and sequenced in parallel to estimate background.

### Western blot

Cells were harvested by lysis in the presence of protease inhibitors, and run on 4–12% Bis-Tris gradient gel (Invitrogen). Protein was transferred onto Nitrocellulose membrane and the membrane was blocked with 5% nonfat dried milk prior to antibody addition. p53 (1:3000, DO1 Santa Cruz), Actin (1:10000, Sigma) antibodies were used. Secondary antibodies with IR-680 (1:10000, Licor) were used for detection.

### ChIP-seq data analysis

All DNA reads in our dataset were single end Illumina reads and were aligned to HG19 genome build using bowtie [40]. Reads were aligned to the HG19 genome with Bowtie1.1 [40], and analyzed with HOMER [41], MACS2 [42] and custom Matlab scripts. Peak calling was done after pooling reads (5-15 M per line, ~ 150 M total) from ChIP-seq experiments in all cell lines. The final set of peaks (8742 peaks) represented the consensus of HOMER (default settings) and MACS2 (using the q < 0.01 threshold) identified peaks, and was filtered to remove ENCODE black-list locations. The number of reads within each peak region was computed from HOMER tag files using custom Matlab (Mathworks) scripts. Background regions around each peak were subtracted from peak scores to correct for high background regions. For each ChIP-seq dataset in our study, the number of reads in p53 peaks was normalized to the average of all cell lines, and for subsequent analyses and comparisons, peaks with less than 2 normalized counts were discarded. We report of the coordinates of these 8742 peaks in Additional file 2: Table S2, together with the normalized read counts for each cell line from our and published datasets (listed below under ‘Published p53 ChIP-seq datasets’ and in Additional file 1: Table S1). Peak numbers in each in each individual dataset (Additional file 1: Table S1) were calculated by determining the fraction of peaks in each cell line with more than 4 normalized counts at a given peak location.

The HOMER package [41] was used for de novo motif discovery. WebLogo was used to generate the motif plot [43] in (Figs. 1, 2b, c) for the top enriched motif. The top enriched motif (Fig. 1b) was then used to re-scan and score all peaks and background regions. Background regions were generated by selecting 500 bp regions adjacent to either side of the peak and excluding regions that overlap with p53 peak regions. Clustering of peaks was accomplished using a Pearson correlation distance metric and average linkage in Matlab.

### RNA-seq data analysis

RNA data was aligned to the Refseq HG19 transcriptome using Tophat, CuffQuant, and CuffMerg [44] or Salmon [45]. Genomic binding and signals were visualized using the UCSC genome browser [46]. Motif analysis was performed in Matlab on the HG19 genome using a ChIP-seq derived PWM adjusted to have a minimum probability of occurrence for each nucleotide.

### Published p53 ChIP-seq datasets

The following p53 ChIP-seq datasets were downloaded from the Sequence Read Archive in the format of raw fastq files using NCBI SRAtools:

- SRR048928, SRR048929 – U2OS cells: Actinomycin D (ActD, 24 h) or Etoposide (Etop, 24 h) treated [7]
- SRR1409975 – HCT116, 5FU (12 h) treated [47]
- SRR287798, SRR287799, SRR287800 – MCF7: RITA (8 h), 5FU (8 h), Nutlin3A (8 h) treated [10]
- SRR575904, SRR575905 – hESC: Doxorubicin (Doxo, 6 h) or Retinoic Acid (RA, 2 d) treated [8]
- SRR851807, SRR851811 – LCL, Doxorubicin (Doxo, 18 h) or IR (4 h) treated [48]
- ERR375900 – CAL51: IR treated (2 h) [49]
- SRR1193314 – BJ: IR treated (6 h) [50]
- SRR1539836 – HCT116, IR treated (8 h) [51]

These datasets were downloaded as raw fastq files and are all single end Illumina reads. Reads were aligned to the HG19 genome with using the same pipeline as described above for our ChIP-seq samples, and further analyzed with HOMER to generate tag files. Custom Matlab code was used to compare these datasets and calculate p53 occupancy in the 8742 peaks identified in our ChIP-seq data (reported in Additional file 2: Table S2).

### Statistics

Statistics relating to motif enrichment or GO-term enrichment were from multiple hypothesis corrected hypergeometric tests performed by HOMER (for motif calling) or using Matlab. Correlation coefficients are Pearson unless otherwise noted and were assigned *p*-values by MATLAB using a two tailed t-test as sample sizes were sufficiently large (1000s).

## Supplementary information

**Additional file 1: Table S1.** p53 ChIP data generated in this study and the public datasets used (in Additional file 4: Figure S1). For each cell line and condition, we also individually called peaks and report the number of peaks identified in each condition.

**Additional file 2: Table S2.** Table of 8742 p53 ChIP peaks reported in this study. Table includes chromosome number, peak start and end coordinates, whether the peak was variably bound across 12 cell lines in this study (494 peaks marked by ‘1’ in the ‘variable peak’ column), the nearest associated gene to each peak as well as the normalized peak intensities for each cell line in this study and in published datasets (Additional file 4: Figure S1, Additional file 1: Table S1).

**Additional file 3: Table S3.** List of p53 target genes used for analysis in Additional file 4: Figure S4. This list was based on [12] and removing genes with low read coverage in our dataset.

**Additional file 4: Figure S1.** Comparison of p53 DNA binding across cell lines and published p53 ChIP-seq datasets. Heatmap showing p53 binding intensity in 8742 locations in the genome in 12 IR treated cell lines (our data, same as Fig. 1c) as well as published datasets (listed in Methods and Table S1). **Figure S2.** Comparison of p53 levels between IR and Nutlin3A treatments. p53 levels were detected by western blot in MCF7 and UACC257 cells. Cells were either untreated, treated with 5 μM Nutlin3A or 4Gy IR for 2 h. **Figure S3.** Reproducibility of in vitro measurements of p53 DNA binding and comparison with in vivo binding. (A) Quantitative agreement in binding strength at p53 binding sites between two replicate p53 in vitro IP datasets using different p53 protein preps. (B) UCSC browser shots of three key binding sites for p53 showing agreement between in vitro binding datasets and divergence with in vivo data. **Figure S4.** Basal gene expression, but not DNA damage induced gene expression, correlates with cell-type specific p53 DNA binding. (A) Boxplots showing the distribution of Pearson’s correlation coefficients of either basal or DNA damage induced fold change of gene expression with p53 binding at the p53 target or variable p53 binding gene sets. (B) Box plots of the fold change of three canonical p53 target genes 3 h after IR. Each dot represents a cell line. CDKN1A/p21 is induced in all cell lines, while MDM2 and BBC3/Puma are cell line dependent. **Figure S5.** p53 binds to IL1A and IL1B in mesenchymal cell lines. UCSC browser screen shot of the p53 ChIP signal. In A549 cells, p53 binds in the proximity of IL1A/IL1B only after TGFβ treatment. Binding of p53 in this region can also be observed in another mesenchymal, CAL51, but not epithelial, HCT116, cell line. **Figure S6.** Knockdown of p53 in LOXIMVI cells reduces expression of inflammatory genes. Expression of p53, IL1A, IL1B, and CXCL1 by qPCR in cells treated with p53 siRNA compared to control siRNA (*N* = 4).

## Data Availability

All sequencing data has been deposited in NCBI’s Gene Expression Omnibus under accession number GSE100292. Data is also available as UCSC tracks as a custom session accessible at: https://genome.ucsc.edu/s/JacobSO/Hafner_et_al_hg19_2019B

## Abbreviations

ATAC-seq: Assay for transposase-accessible chromatin using sequencing
ChIP-seq: Chromatin immunoprecipitation followed by sequencing
EMT: Epithelial to mesenchymal cell transition
IR: Ionizing radiation

## References

[1] Kruiswijk F, Labuschagne CF, Vousden KH (2015). p53 in survival, death and metabolic health: a lifeguard with a licence to kill. Nat Rev Mol Cell Biol.

[2] Hafner A, Bulyk ML, Jambhekar A, Lahav G (2019). The multiple mechanisms that regulate p53 activity and cell fate. Nat Rev Mol Cell Biol.

[3] Fei P, Bernhard EJ, El-Deiry WS (2002). Tissue-specific induction of p53 targets in vivo. Cancer Res.

[4] Brady CA, Attardi LD (2010). p53 at a glance. J Cell Sci.

[5] Aylon Y, Oren M: The Paradox of p53: What, How, and Why? Cold Spring Harbor perspectives in medicine 2016, 6(10). 10.1101/cshperspect.a026328 PMC5046691 27413116

[6] Botcheva K, McCorkle SR, McCombie WR, Dunn JJ, Anderson CW (2011). Distinct p53 genomic binding patterns in normal and cancer-derived human cells. Cell cycle (Georgetown, Tex).

[7] Smeenk L, van Heeringen SJ, Koeppel M, Gilbert B, Janssen-Megens E, Stunnenberg HG, Lohrum M (2011). Role of p53 serine 46 in p53 target gene regulation. PLoS One.

[8] Akdemir KC, Jain AK, Allton K, Aronow B, Xu X, Cooney AJ, Li W, Barton MC (2014). Genome-wide profiling reveals stimulus-specific functions of p53 during differentiation and DNA damage of human embryonic stem cells. Nucleic Acids Res.

[9] Karsli Uzunbas G, Ahmed F, Sammons MA (2019). Control of p53-dependent transcription and enhancer activity by the p53 family member p63. J Biol Chem.

[10] Nikulenkov F, Spinnler C, Li H, Tonelli C, Shi Y, Turunen M, Kivioja T, Ignatiev I, Kel A, Taipale J (2012). Insights into p53 transcriptional function via genome-wide chromatin occupancy and gene expression analysis. Cell Death Differ.

[11] Verfaillie A, Svetlichnyy D, Imrichova H, Davie K, Fiers M, Kalender Atak Z, Hulselmans G, Christiaens V, Aerts S (2016). Multiplex enhancer-reporter assays uncover unsophisticated TP53 enhancer logic. Genome Res.

[12] Fischer M. Census and evaluation of p53 target genes. Oncogene. 2017. 10.1038/onc.2016.502 PMC5511239 28288132

[13] Stewart-Ornstein Jacob, Lahav Galit (2017). p53 dynamics in response to DNA damage vary across cell lines and are shaped by efficiency of DNA repair and activity of the kinase ATM. Science Signaling.

[14] El-Deiry WS, Kern SE, Pietenpol JA, Kinzler KW, Vogelstein B (1992). Definition of a consensus binding site for p53. Nat Genet.

[15] Vassilev LT, Vu BT, Graves B, Carvajal D, Podlaski F, Filipovic Z, Kong N, Kammlott U, Lukacs C, Klein C (2004). In vivo activation of the p53 pathway by small-molecule antagonists of MDM2. Science (New York, NY).

[16] Nguyen TT, Grimm SA, Bushel PR, Li J, Li Y, Bennett BD, Lavender CA, Ward JM, Fargo DC, Anderson CW (2018). Revealing a human p53 universe. Nucleic Acids Res.

[17] Catizone AN, Good CR, Alexander KA, Berger SL, Sammons MA (2019). Comparison of genotoxic versus nongenotoxic stabilization of p53 provides insight into parallel stress-responsive transcriptional networks. Cell cycle (Georgetown, Tex).

[18] Bartlett A, O'Malley RC, Huang SC, Galli M, Nery JR, Gallavotti A, Ecker JR (2017). Mapping genome-wide transcription-factor binding sites using DAP-seq. Nat Protoc.

[19] Jiang S, Mortazavi A (2018). Integrating ChIP-seq with other functional genomics data. Brief Funct Genomics.

[20] Tebaldi T, Zaccara S, Alessandrini F, Bisio A, Ciribilli Y, Inga A (2015). Whole-genome cartography of p53 response elements ranked on transactivation potential. BMC Genomics.

[21] Allen MA, Andrysik Z, Dengler VL, Mellert HS, Guarnieri A, Freeman JA, Sullivan KD, Galbraith MD, Luo X, Kraus WL (2014). Global analysis of p53-regulated transcription identifies its direct targets and unexpected regulatory mechanisms. eLife.

[22] Su D, Wang X, Campbell MR, Song L, Safi A, Crawford GE, Bell DA (2015). Interactions of chromatin context, binding site sequence content, and sequence evolution in stress-induced p53 occupancy and transactivation. PLoS Genet.

[23] ENCODE (2012). An integrated encyclopedia of DNA elements in the human genome. Nature.

[24] Zhong S, He X, Bar-Joseph Z (2013). Predicting tissue specific transcription factor binding sites. BMC Genomics.

[25] Ponnaluri VKC, Zhang G, Esteve PO, Spracklin G, Sian S, Xu SY, Benoukraf T, Pradhan S (2017). NicE-seq: high resolution open chromatin profiling. Genome Biol.

[26] Viswanathan VS, Ryan MJ, Dhruv HD, Gill S, Eichhoff OM, Seashore-Ludlow B, Kaffenberger SD, Eaton JK, Shimada K, Aguirre AJ (2017). Dependency of a therapy-resistant state of cancer cells on a lipid peroxidase pathway. Nature.

[27] Kasai H, Allen JT, Mason RM, Kamimura T, Zhang Z (2005). TGF-beta1 induces human alveolar epithelial to mesenchymal cell transition (EMT). Respir Res.

[28] Gudkov AV, Komarova EA (2003). The role of p53 in determining sensitivity to radiotherapy. Nat Rev Cancer.

[29] Purvis JE, Karhohs KW, Mock C, Batchelor E, Loewer A, Lahav G (2012). p53 dynamics control cell fate. Science (New York, NY).

[30] Paek AL, Liu JC, Loewer A, Forrester WC, Lahav G (2016). Cell-to-cell variation in p53 dynamics leads to fractional killing. Cell.

[31] Sullivan KD, Padilla-Just N, Henry RE, Porter CC, Kim J, Tentler JJ, Eckhardt SG, Tan AC, DeGregori J, Espinosa JM (2012). ATM and MET kinases are synthetic lethal with nongenotoxic activation of p53. Nat Chem Biol.

[32] John S, Sabo PJ, Thurman RE, Sung MH, Biddie SC, Johnson TA, Hager GL, Stamatoyannopoulos JA (2011). Chromatin accessibility pre-determines glucocorticoid receptor binding patterns. Nat Genet.

[33] Lidor Nili E, Field Y, Lubling Y, Widom J, Oren M, Segal E (2010). p53 binds preferentially to genomic regions with high DNA-encoded nucleosome occupancy. Genome Res.

[34] Lowe JM, Menendez D, Bushel PR, Shatz M, Kirk EL, Troester MA, Garantziotis S, Fessler MB, Resnick MA (2014). p53 and NF-kappaB coregulate proinflammatory gene responses in human macrophages. Cancer Res.

[35] Arandkar S, Furth N, Elisha Y, Nataraj NB, van der Kuip H, Yarden Y, Aulitzky W, Ulitsky I, Geiger B, Oren M (2018). Altered p53 functionality in cancer-associated fibroblasts contributes to their cancer-supporting features. Proc Natl Acad Sci U S A.

[36] Reyes José, Chen Jia-Yun, Stewart-Ornstein Jacob, Karhohs Kyle W., Mock Caroline S., Lahav Galit (2018). Fluctuations in p53 Signaling Allow Escape from Cell-Cycle Arrest. Molecular Cell.

[37] Hafner A, Stewart-Ornstein J, Purvis JE, Forrester WC, Bulyk ML, Lahav G (2017). p53 pulses lead to distinct patterns of gene expression albeit similar DNA-binding dynamics. Nat Struct Mol Biol.

[38] Martin M (2011). Cutadapt removes adapter sequences from high-throughput sequencing reads. EMBnetjournal.

[39] Buenrostro JD, Giresi PG, Zaba LC, Chang HY, Greenleaf WJ (2013). Transposition of native chromatin for fast and sensitive epigenomic profiling of open chromatin, DNA-binding proteins and nucleosome position. Nat Methods.

[40] Langmead B, Trapnell C, Pop M, Salzberg SL (2009). Ultrafast and memory-efficient alignment of short DNA sequences to the human genome. Genome Biol.

[41] Heinz S, Benner C, Spann N, Bertolino E, Lin YC, Laslo P, Cheng JX, Murre C, Singh H, Glass CK (2010). Simple combinations of lineage-determining transcription factors prime cis-regulatory elements required for macrophage and B cell identities. Mol Cell.

[42] Zhang Y, Liu T, Meyer CA, Eeckhoute J, Johnson DS, Bernstein BE, Nusbaum C, Myers RM, Brown M, Li W (2008). Model-based analysis of ChIP-Seq (MACS). Genome Biol.

[43] Crooks GE, Hon G, Chandonia JM, Brenner SE (2004). WebLogo: a sequence logo generator. Genome Res.

[44] Trapnell C, Roberts A, Goff L, Pertea G, Kim D, Kelley DR, Pimentel H, Salzberg SL, Rinn JL, Pachter L (2012). Differential gene and transcript expression analysis of RNA-seq experiments with TopHat and cufflinks. Nat Protoc.

[45] Patro R, Duggal G, Love MI, Irizarry RA, Kingsford C (2017). Salmon provides fast and bias-aware quantification of transcript expression. Nat Methods.

[46] Kent WJ, Sugnet CW, Furey TS, Roskin KM, Pringle TH, Zahler AM, Haussler D (2002). The human genome browser at UCSC. Genome Res.

[47] Sanchez Y, Segura V, Marin-Bejar O, Athie A, Marchese FP, Gonzalez J, Bujanda L, Guo S, Matheu A, Huarte M (2014). Genome-wide analysis of the human p53 transcriptional network unveils a lncRNA tumour suppressor signature. Nat Commun.

[48] Zeron-Medina J, Wang X, Repapi E, Campbell MR, Su D, Castro-Giner F, Davies B, Peterse EF, Sacilotto N, Walker GJ (2013). A polymorphic p53 response element in KIT ligand influences cancer risk and has undergone natural selection. Cell.

[49] Rashi-Elkeles S, Warnatz HJ, Elkon R, Kupershtein A, Chobod Y, Paz A, Amstislavskiy V, Sultan M, Safer H, Nietfeld W (2014). Parallel profiling of the transcriptome, cistrome, and epigenome in the cellular response to ionizing radiation. Sci Signal.

[50] Williams K, Christensen J, Rappsilber J, Nielsen AL, Johansen JV, Helin K (2014). The histone lysine demethylase JMJD3/KDM6B is recruited to p53 bound promoters and enhancer elements in a p53 dependent manner. PLoS One.

[51] Desantis A, Bruno T, Catena V, De Nicola F, Goeman F, Iezzi S, Sorino C, Gentileschi MP, Germoni S, Monteleone V (2015). Che-1 modulates the decision between cell cycle arrest and apoptosis by its binding to p53. Cell Death Dis.

