# Supplementary material for: Identification of universal and cell-type specific p53 DNA binding
Source: BMC Mol Cell Biol. 2020 Feb 18;21:5. doi: 10.1186/s12860-020-00251-8 (PMC7027055; doi:10.1186/s12860-020-00251-8)
Supplement: Supplementary file 3 — Additional file 3: Table S3. List of p53 target genes used for analysis in Additional file 4: Figure S4. This list was based on [12] and removing genes with low read coverage in our dataset. [file 12860_2020_251_MOESM3_ESM.rtf]

p53 Target Genes used in this analysisAENANKRA2ANXA4APOBEC3CASCC3BAXBBC3BLOC1S2BTG2CCDC90BCCNG1CDKN1ACERS5CES2CMBLCSF1CYFIP2DCP1BDDB2DRAM1DUSP14DYRK3EPHA2EPS8L2FAM210BFBXO22FDXRFOSL1FUCA1GADD45AGDF15HSPA4LIER5IKBIPISCUKITLGLIFMDM2MICALL1NADSYN1NINJ1NTPCRORAI3PGPEP1PHLDA3PLK2PLK3PMAIP1POLHPPM1DPRKAB1PTP4A1RAP2BRNF19BRPS27LRRM2BSAC3D1SERTAD1SESN1SESN2SLC12A4SLC30A1SULF2SUSD6TGFATM7SF3TMEM68TNFRSF10BTNFRSF10DTP53I3TRAF4TRIAP1XPCZNF219ZNF337ZNF79
