# Supplementary material for: Identification of universal and cell-type specific p53 DNA binding
Source: BMC Mol Cell Biol. 2020 Feb 18;21:5. doi: 10.1186/s12860-020-00251-8 (PMC7027055; doi:10.1186/s12860-020-00251-8)
Supplement: Supplementary file 4 — Additional file 4: Figure S1. Comparison of p53 DNA binding across cell lines and published p53 ChIP-seq datasets. Heatmap showing p53 binding intensity in 8742 locations in the genome in 12 IR treated cell lines (our data, same as Fig. 1c) as well as published datasets (listed in Methods and Table S1). Figure S2. Comparison of p53 levels between IR and Nutlin3A treatments. p53 levels were detected by western blot in MCF7 and UACC257 cells. Cells were either untreated, treated with 5 μM Nutlin3A or 4Gy IR for 2 h. Figure S3. Reproducibility of in vitro measurements of p53 DNA binding and comparison with in vivo binding. (A) Quantitative agreement in binding strength at p53 binding sites between two replicate p53 in vitro IP datasets using different p53 protein preps. (B) UCSC browser shots of three key binding sites for p53 showing agreement between in vitro binding datasets and divergence with in vivo data. Figure S4. Basal gene expression, but not DNA damage induced gene expression, correlates with cell-type specific p53 DNA binding. (A) Boxplots showing the distribution of Pearson’s correlation coefficients of either basal or DNA damage induced fold change of gene expression with p53 binding at the p53 target or variable p53 binding gene sets. (B) Box plots of the fold change of three canonical p53 target genes 3 h after IR. Each dot represents a cell line. CDKN1A/p21 is induced in all cell lines, while MDM2 and BBC3/Puma are cell line dependent. Figure S5. p53 binds to IL1A and IL1B in mesenchymal cell lines. UCSC browser screen shot of the p53 ChIP signal. In A549 cells, p53 binds in the proximity of IL1A/IL1B only after TGFβ treatment. Binding of p53 in this region can also be observed in another mesenchymal, CAL51, but not epithelial, HCT116, cell line. Figure S6. Knockdown of p53 in LOXIMVI cells reduces expression of inflammatory genes. Expression of p53, IL1A, IL1B, and CXCL1 by qPCR in cells treated with p53 siRNA compared to control siRNA (N = 4). [file 12860_2020_251_MOESM4_ESM.pdf]

Figure S1

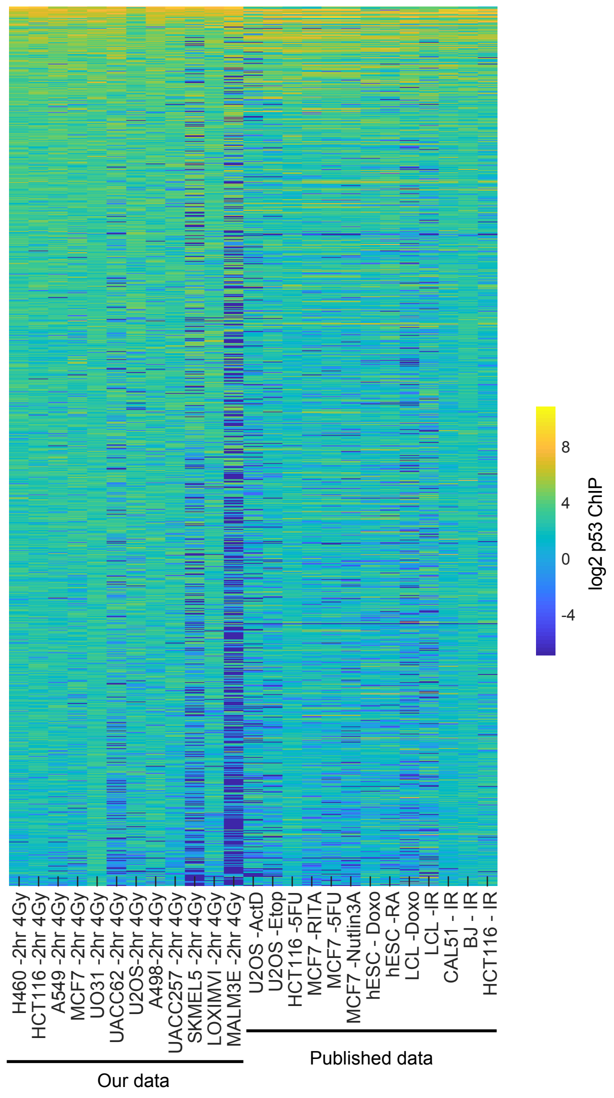

Figure S1: Comparison of p53 DNA binding across cell lines and published p53 ChIP-seq datasets. Heatmap showing p53 binding intensity in 8742 locations in the genome in 12 IR treated cell lines (our data, same as Fig. 1C) as well as published datasets (listed in Methods and Table S1).

Figure S2

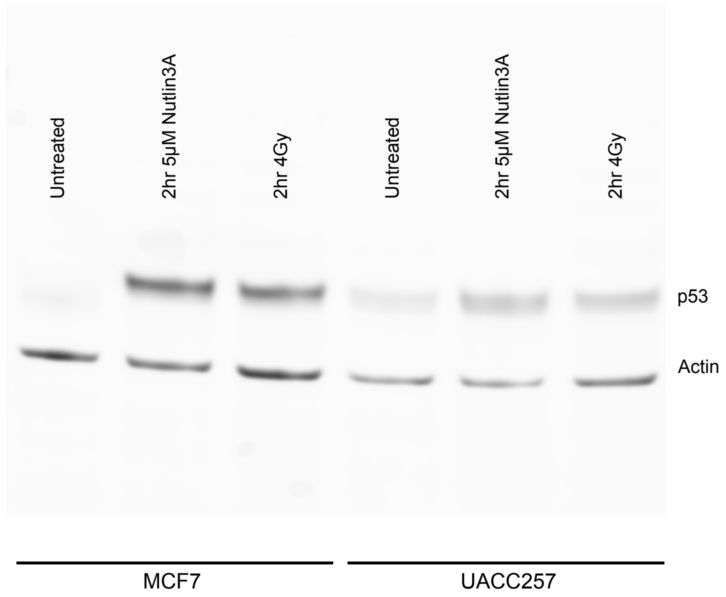

Figure S2: Comparison of p53 levels between IR and Nutlin3A treatments. p53 levels were detected by western blot in MCF7 and UACC257 cells. Cells were either untreated, treated with 5µM Nutlin3A or 4Gy IR for 2 hours.

Figure S3

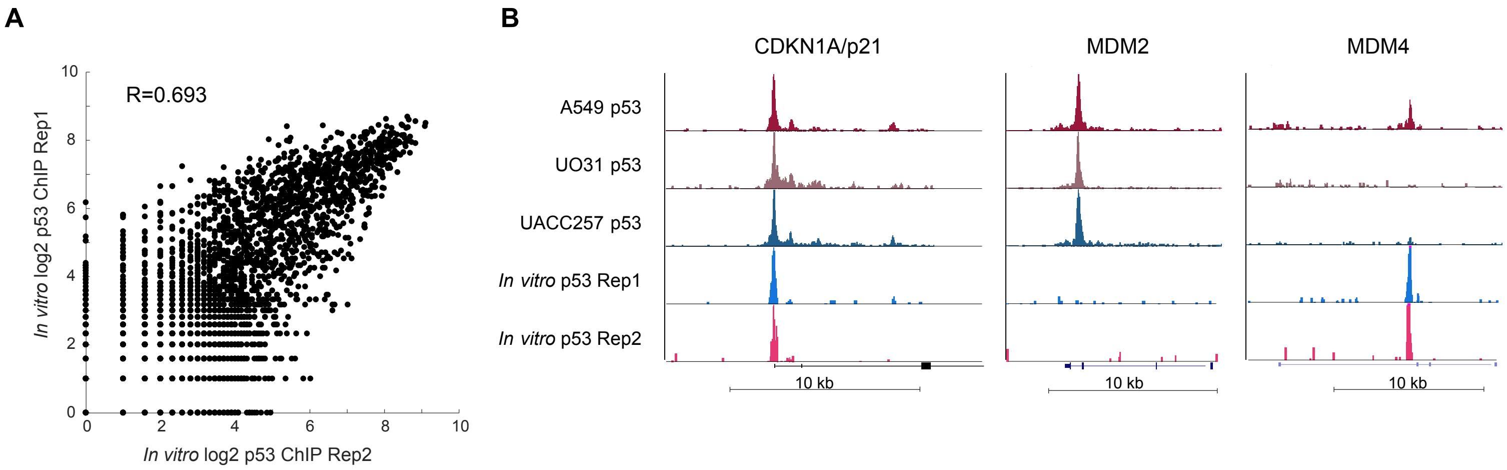

Figure S3: Reproducibility of in vitro measurements of p53 DNA binding and comparison with in vivo binding. (A) Quantitative agreement in binding strength at p53 binding sites between two replicate p53 in vitro IP datasets using different p53 protein preps. (B) UCSC browser shots of three key binding sites for p53 showing agreement between *in vitro* binding datasets and divergence with *in vivo* data.

Figure S4

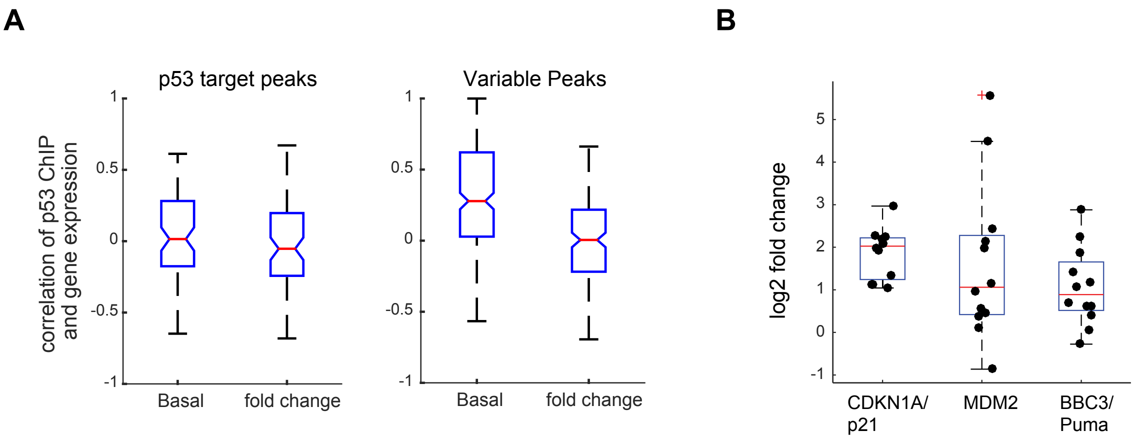

Figure S4: Basal gene expression, but not DNA damage induced gene expression, correlates with cell-type specific p53 DNA binding. (A) Boxplots showing the distribution of Pearson's correlation coefficients of either basal or DNA damage induced fold change of gene expression with p53 binding at the p53 target or variable p53 binding gene sets. (B) Box plots of the fold change of three canonical p53 target genes 3h after IR. Each dot represents a cell line. CDKN1A/p21 is induced in all cell lines, while MDM2 and BBC3/Puma are cell line dependent.

Figure S5

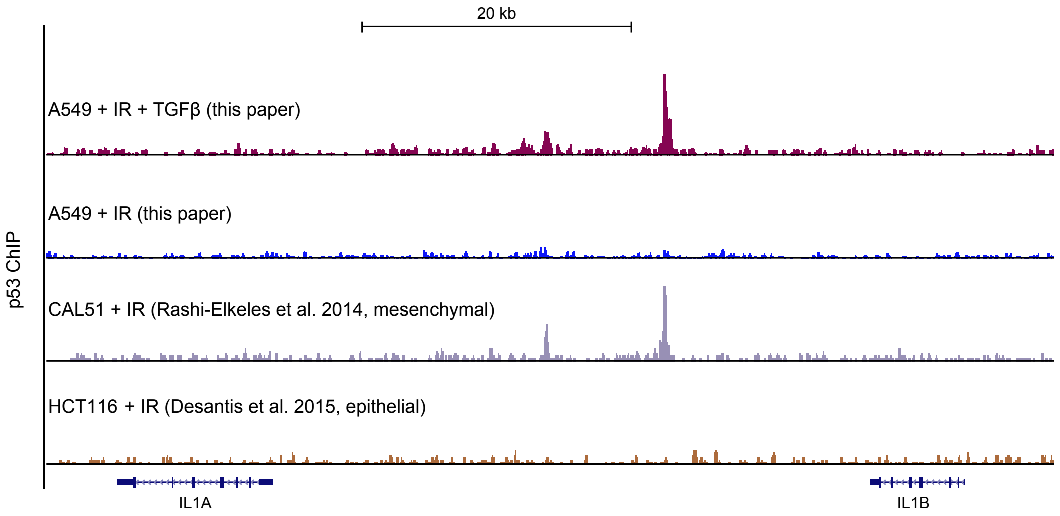

Figure S5: p53 binds to IL1A and IL1B in mesenchymal cell lines. UCSC browser screen shot of the p53 ChIP signal. In A549 cells, p53 binds in the proximity of IL1A/IL1B only after TGFβ treatment. Binding of p53 in this region can also be observed in another mesenchymal, CAL51, but not epithelial, HCT116, cell line.

Figure S6

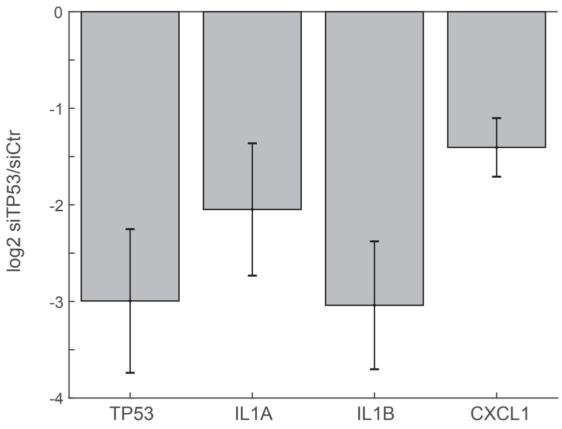

Figure S6: Knockdown of p53 in LOXIMVI cells reduces expression of inflammatory genes. Expression of p53, IL1A, IL1B, and CXCL1 by qPCR in cells treated with p53 siRNA compared to control siRNA (N=4).
